# Canady Helios Cold Plasma Induces Non-Thermal (24 °C), Non-Contact Irreversible Electroporation and Selective Tumor Cell Death at Surgical Margins

**DOI:** 10.3390/cancers17233869

**Published:** 2025-12-02

**Authors:** Saravana R. K. Murthy, Taisen Zhuang, Olivia Z. Jones, Yasmine Dakak, Michael Keidar, Aviram Nissan, Jerome Canady

**Affiliations:** 1 Department of Translational Research, Jerome Canady Research Institute for Advanced Biological and Technological Sciences, Takoma Park, MD 20912, USA; drsmurthy@jcri-abts.com (S.R.K.M.); tzhuang@usmedinnov.com (T.Z.); ozjones@jcri-abts.com (O.Z.J.); yasminedak@gwu.edu (Y.D.); 2 Department of Mechanical and Aerospace Engineering, The George Washington University, Washington, DC 20052, USA; keidar@gwu.edu; 3 Department of Surgery, Ziv Medical Center, Safed 13100, Israel; aviramn@ziv.gov.il; 4 Department of Surgery, University of Maryland, Capital Regional Medical Center, Largo, MD 20774, USA

**Keywords:** plasma treated electromagnetic field (PTEF), irreversible electroporation (IRE), breast cancer, cold atmospheric plasma, cancer treatment, surgical margin treatment, non-contact, non-thermal (24 °C)

## Abstract

Background: The Canady Helios Cold Plasma (CHCP) system is a non-thermal, non-contact cold atmospheric plasma technology that generates transient electric fields and reactive species capable of disrupting cancer cell membranes. This study investigated the voltage-dependent membrane irreversible electroporation (IRE) dynamics induced by CHCP across biologically distinct breast cancer subtypes. Methods: Four breast cancer cell lines, triple-negative (MDA-MB-231 and Hs578T), ER^+^/PR^+^/HER2^−^ (MCF-7), and ER^+^/PR^+^/HER2^+^ (BT-474), were exposed to CHCP for 5 min at 25 V (~1675 V/cm PTEF) or 30 V (~2010 V/cm), either directly or with Plasma Activated Media (PAM). Membrane permeability was assessed by propidium iodide (PI) uptake over 120 min. Morphological changes were evaluated microscopically. Functional electroporation was examined via BCL2A1-targeting *si*RNA delivery and clonogenic survival. Ex vivo analyses of Phase I clinical trial tumor specimens (NCT04267575) were performed to characterize CHCP-induced tissue responses. Results: CHCP produced voltage- and time-dependent membrane permeabilization in all breast cancer cell lines, with 30 V generating robust and sustained PI uptake compared to transient effects at 25 V. Treated cells exhibited morphological features consistent with membrane disruption. CHCP enabled intracellular siRNA delivery and significantly reduced clonogenic potential, confirming functional pore formation. Ex vivo CHCP treatment selectively damaged tumor cells while sparing adjacent non-cancerous tissue. Conclusions: This study demonstrates CHCP as a non-thermal (24 °C), non-contact plasma-based IRE platform which induces controlled membrane permeabilization and selective cancer cell death. CHCP offers a translational strategy to eradicate residual tumor cells at the surgical margins, and prevent local recurrence, positioning it as a versatile adjunct in precision surgical oncology.

## Simple Summary

Residual tumor cells left behind after surgery are a major cause of cancer local recurrence. The Canady Helios Cold Plasma (CHCP) system is a novel non-thermal (24 °C), non-contact technology that generates plasma electromagnetic fields capable of selectively killing cancer cells while sparing surrounding normal tissue. In this study, we demonstrate that CHCP induces irreversible electroporation (IRE), a process that disrupts tumor cell membranes and enables entry of molecules such as dyes and therapeutic siRNAs. Using multiple breast cancer cell lines, CHCP was shown to cause voltage- and time-dependent membrane permeabilization, leading to cell death and loss of tumor-forming ability. Importantly, analysis of tumor margin specimens from our Phase I clinical trial (NCT04267575) confirmed that CHCP effectively killed tumor cells while preserving adjacent healthy tissue. These findings highlight CHCP as the first plasma-based IRE platform to induce selective tumor cell death at the surgical margins, thus reducing local recurrence and improving survival outcomes for patients after surgical resection of solid tumors.

## 1. Introduction

Irreversible electroporation (IRE) is a non-thermal tumor ablation technique that induces nanoscale pores in the plasma membrane, disrupting cellular homeostasis and leading to cell death while preserving extracellular matrix and vascular structures [1]. Conventional IRE systems, such as NanoKnife and Galvanize Knife, deliver high-voltage pulses via direct electrode insertion, which can produce thermal effects and carry procedural risks, including venous thrombosis, biliary complications, gastrointestinal injuries, and rectal fistula, depending on the tumor location [2,3,4]. Moreover, histologic and imaging studies provide only indirect evidence that NanoKnife or similar platforms achieve true non-thermal IRE, highlighting the need for alternative modalities that combine precision, safety, and mechanistic clarity [5]. Traditional thermal-based surgical and ablative modalities also pose risks of collateral tissue damage and delayed healing [6], emphasizing the importance of developing approaches that selectively target malignant cells while sparing surrounding normal tissue.

Cold atmospheric plasma (CAP) has rapidly emerged as a transformative modality in cancer therapy, offering a non-thermal approach through the generation of reactive oxygen and nitrogen species (RONS) that selectively target malignant cells [7]. Over the past decade, our group has pioneered both the preclinical and clinical translation of CAP technologies, culminating in the development of the Canady Helios Cold Plasma (CHCP) system, the first FDA-approved CAP device (K240297) in the United States for intraoperative use [8,9,10,11]. Preclinical studies have demonstrated CAP’s ability to induce apoptosis, disrupt cell cycles [7], and inhibit proliferation in a variety of cancer cell types, including breast [10], head and neck [12], colon, glioblastoma [13], and soft tissue sarcoma [8], while sparing normal tissue. The first Phase I clinical trial of CHCP for advanced solid tumors (NCT04267575) [14] established safety and feasibility, with evidence of effective microscopic tumor cell death at the margin, exceptional local regional recurrence control, and promising survival outcomes without adverse effects [14]. In our published Phase I clinical trial we have explained the scientific background and rationale for the trial [14]. Previous studies showed that applying gas plasma increased the membrane permeability, allowing macromolecules to enter cells and disrupt homeostasis [15]. While conventional IRE relies solely on direct electrode-delivered pulses [16], CHCP integrates a high-frequency electric charge with an inert gas which creates a localized Plasma Treated Electromagnetic Field (PTEF) that includes reactive oxygen and nitrogen species, including Hydrogen peroxide (H_2_O_2_), Atomic oxygen [O(^3^P)], Ozone (O_3_), Singlet oxygen (^1^O_2_), Superoxide anion (^•^O_2_^−^), Carbonate radicals (CO_3_^−^), Nitric oxide (NO^⋅^), Nitrogen dioxide (NO_2_^⋅^), Nitrite (NO_2_^−^), Nitrate (NO_3_^−^), Peroxynitrite (ONOO^−^), and Peroxynitric acid (ONOOH) [14,17]. A PTEF provides a quantitative, reproducible framework for assessing membrane permeabilization during plasma treatment, distinguishing CHCP from tumor treating fields (TTFs) and electrode-based IRE platforms. This unique configuration raises a critical unanswered question: can a non-contact plasma system achieve controlled, voltage-dependent electroporation analogous to traditional IRE? This knowledge gap has impeded mechanistic clarity and limited the positioning of CHCP within the broader IRE and CAP literature.

In this study, we address this gap by systematically evaluating CHCP-induced electroporation across four biologically distinct breast cancer cell lines: triple-negative (MDA-MB-231, Hs578T), ER^+^/PR^+^/HER2^−^ (MCF-7), and ER^+^/PR^+^/HER2^+^ (BT-474). Using propidium iodide (PI) uptake as a sensitive marker of membrane integrity, we quantify the field-strength and time-dependent responses to CHCP treatment. PI is a membrane-impermeant dye that fluoresces upon binding nucleic acids, providing a direct readout of membrane permeabilization and IRE-induced cell death. We further demonstrate the functional effects of CHCP-induced electroporation, that includes intracellular delivery of small interfering RNA (siRNA) targeting the anti-apoptotic gene BCL2A1. We also showed that CHCP induced IRE by suppression of the clonogenic potential of breast cancer cell lines. Moreover, morphological analyses of breast cancer cells and pathological analysis of patient tumor samples obtained from the Phase I Clinical trial (NCT04267575) [14] demonstrated selective induction of tumor cell death after CHCP treatment. Together, these findings establish the CHCP-induced PTEF as a novel, non-thermal (24 °C), and non-contact IRE (NTNC-IRE) platform for selective IRE-based cancer therapy, with a significant translational effect for selective tumor cell death at the surgical margin and recurrence prevention. 

## 2. Materials and Methods

### 2.1. Cold Plasma Device

**Figure 1:**
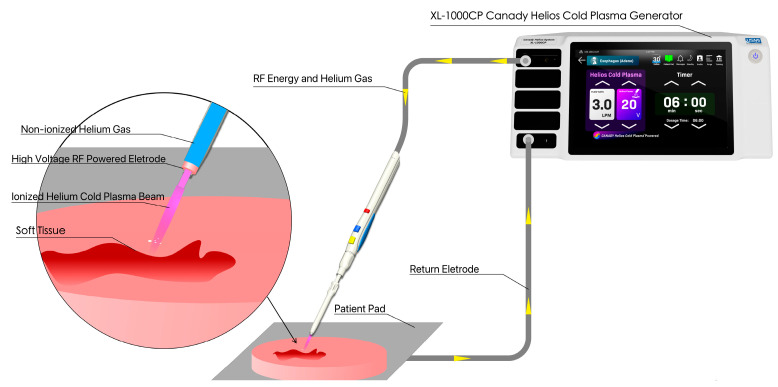
Illustration showing the CHCP system.

The Canady Helios Cold Plasma (CHCP) XL-1000 CP System was used for performing all experiments at the Jerome Canady Research Institute for Advanced Biological and Technological Sciences (JCRI-ABTS), Takoma Park, MD, USA. Briefly, the CHCP XL-1000CP device (FDA cleared in 2024) integrates a high-frequency power generator, helium gas regulation system, and timer functions into a single unit. The system delivers an output voltage of up to 6 kV at a frequency of approximately 300 kHz, with a power output of less than 40 W. The CHCP system is designed to operate with the FDA-cleared Canady Helios Cold Plasma Ablator, a disposable handpiece used for the ablation of tumor tissue. Details and schematics on the plasma generation by CHCP used in this study are shown in Figure 1. The helium flow rate was set to a constant 3 L/min and the applied voltage was set to 25 V (~1675 V/cm PTEF) and 30 V (~2010 V/cm PTEF). The plasma jet nozzle was positioned 1.5 cm above the media surface (~2.0 cm above the adherent cell layer). The applied voltage was set to 15–30 V RMS (corresponding to ~1005–2010 V/cm field strength). The system output of up to 6 kV refers to the maximum internal discharge potential, not the applied treatment voltage. The plasma scalpel tip was placed 1.5 cm above the surface of the cell media and remained unmoved for the duration of the treatment. The CAP treatment was performed in a laminar airflow tissue culture hood, Purifier Logic + Class II, Type A2 Biosafety Cabinet (Labconco, Kansas City, MO, USA) at room temperature.

### 2.2. Definition and Calculation of the Plasma Treated Electromagnetic Field (PTEF)

To quantitatively characterize the electrical component of CHCP treatment, we define the Plasma Treated Electromagnetic Field (PTEF) as the effective electric field strength generated between the CHCP central electrode and the return ground pad beneath the cell culture. The PTEF field strength was calculated using the following equation:

$$F=\frac{{\gamma \times V}_{in}}{D}$$

where $V_{in}$ is the input voltage supplied by the CHCP XL-1000 CP generator, γ is the voltage conversion coefficient (experimentally determined as 100), and D is the fixed distance between the plasma electrode tip and the cell culture surface (1.5 cm in this study). PTEF Field Strength used in this study is shown in Table 1. It should be noted that the reported values represent average electric field strengths; the peak electric field may be higher [18].

**Figure 2:**
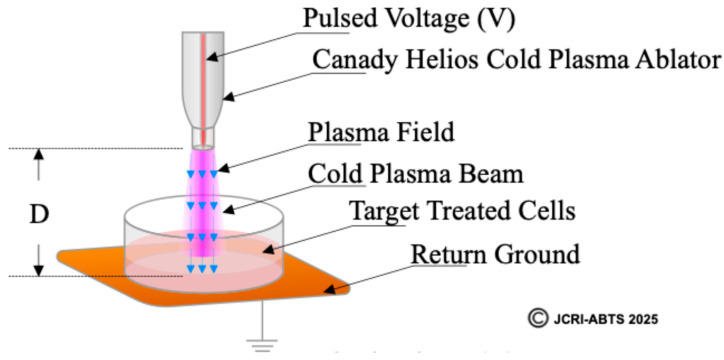
Schematic representation of the Plasma Treated Electromagnetic Field (PTEF) generated by the Canady Helios Cold Plasma (CHCP) system.

A schematic illustration of the PTEF (Figure 2) shows the relationship between the plasma electrode, target cells, and ground pad, with the field vector indicated by a blue arrow. This figure provides a visual definition of the PTEF and its experimental geometry. The top is highlighting the Canady Helios Cold Plasma Ablator^TM^, the plasma beam directed toward the cell culture, the fixed distance (D), and the return ground. This diagram visually defines the PTEF as the spatially directed electric field vector that integrates both the localized electromagnetic field and the plasma-generated species to induce membrane permeabilization in target cells.

### 2.3. Cell Culture

Cell culture experiments were carried out as described previously. The human breast cancer cell line BT-474 was purchased from ATCC (Manassas, VA, USA). MCF-7, MDA-MB-231, and Hs578T were generously donated by Professor Kanaan’s laboratory at Howard University. All cell lines were cultured in Roswell Park Memorial Institute (RPMI) 1640 Medium supplemented with 10% fetal bovine serum (Sigma-Aldrich, St. Louis, MO, USA) and 1% Pen Strep (Thermo Fisher Scientific, Waltham, MA, USA) in a 37 °C and 5% CO2 humidified incubator (Thermo Fisher Scientific, Waltham, MA, USA). When cells reached approximately 80% confluence, cells were seeded at a concentration of 10^5^ cells/well into 12-well plates (USA Scientific, Ocala, FL, USA) with a 1 mL media volume per well for all experiments.

### 2.4. Treatment Protocol

Cells were directly treated with CHCP at 15 V, 20 V, 25 V (~1675 V/cm PTEF), and 30 V (1005–2010 V/cm) for 5 min using helium as the carrier gas or with plasma-activated media (PAM). The plasma jet nozzle was positioned 1.5 cm above the media surface, which corresponded to ~2.0 cm above the adherent cell layer. Helium-treated cells served as negative controls. In this study, the “input voltage” (Vin) refers to the generator control setting applied to the full-bridge converter stage of the CHCP power supply, not the actual plasma output voltage. This control parameter (e.g., 25 V or 30 V) determines the field strength through proportional adjustment of the internal high-voltage transformer and is therefore used as the practical control variable for defining the Plasma Treatment Electrical Field (PTEF). The system’s maximum output capability is 6 kV; however, the control voltage is reported here for consistency with our experimental setup and calibration curve (gamma = 100).

### 2.5. Electroporation Assay and Propidium Iodide Staining 

Human breast cancer cell lines (Hs578T, BT-474, MCF-7, and MDA-MB-231) were seeded at a density of 1 × 10^5^ cells per well in 12-well tissue culture plates and incubated overnight at 37 °C in a humidified atmosphere containing 5% CO_2_. Cells were treated with CHCP using a standardized system, as explained above. Untreated and helium-only exposed cells served as controls. Post-treatment, cells were incubated for 0, 30, 60, and 120 min, followed by staining with propidium iodide (PI). The PI uptake intensity, indicating membrane permeability, was quantified via fluorescence imaging and analyzed statistically (*n* = 3 per condition). Briefly, the culture medium was removed, and cells were incubated with propidium iodide (PI; 1–5 μg/mL in phenol red-free RPMI medium) for 30 min at 37 °C, protected from light. PI selectively stains cells with compromised membranes by intercalating into DNA, emitting fluorescence upon binding (excitation: 535 nm; emission: 617 nm). After incubation, cells were gently washed with phosphate-buffered saline (PBS) to remove unbound dye and subsequently fixed with 4% paraformaldehyde for 10 min or formalin for 5 min at room temperature. Cells were then washed again with PBS. Fluorescence intensity was quantified using a microplate reader equipped with appropriate filters (excitation at 535 nm and emission at 617 nm) (BioTek Synergy HTX (Winooski, VT, USA)). The fluorescence signal corresponds to the proportion of membrane-compromised cells. Fluorescence data from the treated and control groups were analyzed to assess the plasma-induced membrane permeability. Statistical significance was determined using appropriate tests (e.g., Student’s *t*-test or ANOVA), with *p*-values < 0.05 considered significant. The data was plotted by Microsoft Excel 2024 (Redmond, WA, USA) as mean ± standard error of the mean.

### 2.6. Transfection of siRNA 

MISSION^®^ esiRNA targeting human BCL2A1 and a non-targeting scrambled control were obtained from Sigma-Aldrich. MCF-7, MDA-MB-231, and Hs578T cells were seeded in 12-well plates at 1 × 10^5^ cells/well and cultured overnight in RPMI-1640 medium supplemented with 10% FBS and 1% Penicillin–Streptomycin at 37 °C and 5% CO_2_. After 24 h, cells were exposed to CHCP as described above. Thirty minutes after treatment, the medium was replaced with antibiotic-free RPMI containing either 13 pmol/mL BCL2A1 esiRNA or scrambled esiRNA. Cells were incubated for 6 h, after which the total RNA was isolated for downstream analysis.

### 2.7. Quantitative Real-Time RT-PCR

Total RNA was extracted from MCF-7, MDA-MB-231, and Hs578T cells using TRI Reagent followed by the Direct-zol MiniPrep Kit (Zymo Research, Irvine, CA, USA) with on-column DNase treatment. First-strand cDNA was synthesized from 1 µg RNA using the Transcriptor First Strand cDNA Synthesis Kit (Roche Applied Science, Penzberg, Germany). qRT-PCR was performed according to MIQE guidelines using SYBR Green Master Mix (Applied Biosystems, Waltham, MA, USA). Each reaction contained 1 µL of cDNA (1:20 dilution). The cycling conditions were as follows: 95 °C for 15 s, 60 °C for 60 s, and 72 °C for 30 s for 40 cycles, followed by a 10 min extension at 72 °C. Expression levels were normalized to 18S rRNA, and relative changes were calculated using the 2−^ΔΔCT^ method.

### 2.8. Plate Colony Formation Assay

MDA-MB-231, MCF-7, and Hs578T cells were seeded at a density of 1 × 10^5^ cells per well in 6-well culture plates and incubated for 24 h under standard culture conditions (37 °C, 5% CO_2_). Cells were subsequently treated with CHCP at voltages of 15 V, 20 V, 25 V, or 30 V for 5 min. Following treatment, cells were harvested at three different post-treatment incubation times (30, 60, and 120 min), diluted 1:1000 in fresh RPMI medium, and reseeded into 12-well plates to allow colony formation. After 7–10 days of incubation, colonies were fixed with 10% neutral-buffered formalin for 30 min and stained with 1% crystal violet for 15 min. Excess dye was removed by washing three times with phosphate buffered saline (PBS), and plates were air-dried at room temperature. Colony formation was quantified by counting visible colonies containing ≥50 cells under a light microscope. Colony numbers were recorded as mean ± SEM from three independent experiments.

### 2.9. Patient Samples

The Phase I clinical trial (NCT04267575) information of participants, interventions, objectives, outcomes, sample size, assignment method, unit of analysis, and statistical methods were already published in our Phase I clinical trial results publication [1]. In this study involving R0 patients, FFPE sample sections from patients R0009 (metastatic pleomorphic sarcoma of the left distal femur) and R0004 (metastatic recurrent non-small cell lung adenocarcinoma of the left hip/proximal femur) with or without Canady Helios Cold Plasma (CHCP) treatment were used.

### 2.10. Hematoxylin and Eosin (H&E) Staining of Tissue Specimens

Fresh tissue specimens, with or without ex vivo CHCP treatment, were fixed in 10% neutral-buffered formalin for 24–48 h. Fixed tissues were dehydrated through a graded ethanol series, cleared in xylene, and embedded in paraffin wax. Serial sections of 6–7 μm thickness were prepared using a microtome and mounted on glass slides. Sections were deparaffinized in xylene, rehydrated through descending ethanol concentrations, and subjected to standard H&E staining. Briefly, slides were stained with hematoxylin to visualize nuclei, differentiated in acid–alcohol, and counterstained with eosin to highlight cytoplasmic and extracellular components. Following staining, sections were dehydrated with ethanol, and cover-slipped using a permanent mounting medium. Stained slides were examined under a light microscope (63× objective) to assess cellular morphology, tissue architecture, and pathological alterations, including plasma-induced cell death and membrane compromise.

### 2.11. Statistical Analysis

The data are presented as mean ± SEM unless otherwise stated. Each experiment was conducted across three independent days, and each condition included multiple technical replicates per day. The normality of residuals was assessed using the Shapiro–Wilk test, and the homogeneity of variance was evaluated using Levene’s test. The data met the assumptions of normality (Shapiro–Wilk *p* > 0.05 for all comparisons) and homogeneity of variance (Levene’s *p* > 0.05), supporting the use of parametric tests in all the tested data samples unless otherwise mentioned in the Results Section. Pairwise comparisons between CHCP-treated and helium control groups were performed using two-tailed Student’s ***t***-tests. For multi-group analyses, one-way ANOVA was used, followed by Tukey’s post-hoc correction for multiple comparisons. A *p*-value < 0.05 was considered statistically significant.

## 3. Results

### 3.1. CHCP Induces Voltage- and Time-Dependent Electroporation in Breast Cancer Cells

**Figure 3:**
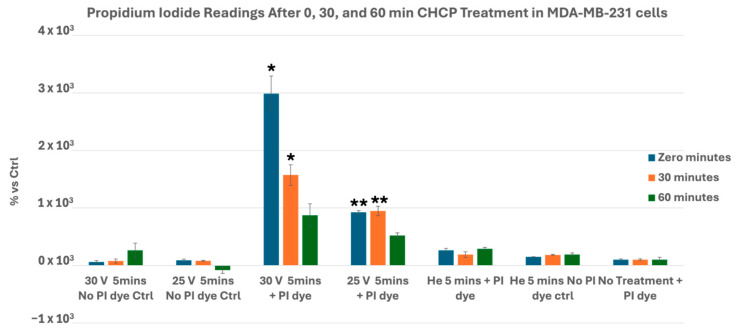
Analysis of electroporation effects of CHCP treatment on triple-negative breast cancer MDA-MB-231 cells using PI intake intensity measurements. Cells were treated with CHCP at 30 V, CHCP at 25 V, or helium for 5 min, followed by analysis at multiple incubation time points (0, 30, and 60 min) to assess the plasma-induced membrane permeability. The percentage of PI intensity indicates the degree of cell membrane electroporation, with the data representing the mean ± SEM from *n* = 3 biological replicates, each containing triplicate technical measurements. Statistical significance was analyzed between CHCP-treated and helium-treated samples using Student’s *t*-test, where at 30 V, at zero, 0.5, and 1 h, it was *p* = 0.016, *p* = 0.020, and *p* = 0.062, respectively. At 25 V, at zero, 0.5, and 1 h, it was *p* = 0.001, *p* =0.008, and *p* = 0.618, respectively. Data are represented by * *p* < 0.05 and ** *p* < 0.01. Although the bar graph for the 30 V group at the 60-min time point appears comparable to or greater than the 25 V group at earlier incubation periods, one of the three replicate measurements for the 30 V 60 min group was similar to the helium control, resulting in higher variance within this dataset. Consequently, statistical significance was not achieved for this time point, and the SEM error bar appears relatively longer in the graph.

To evaluate the electroporation capability of CHCP across breast cancer subtypes, MDA-MB-231, MCF-7, Hs578T, and BT-474 cells were treated with CHCP at 25 V and 30 V for 5 min, and membrane permeability was quantified by propidium iodide (PI) uptake at 0, 30, and 60 min post-treatment. CHCP treatment induced clear voltage-dependent electroporation in MDA-MB-231 cells, as reflected by PI uptake kinetics. At 30 V (~2010 V/cm PTEF), membrane permeability was significantly elevated immediately (*p* = 0.016) and at 30 min (*p* = 0.020), with partial recovery by 60 min (*p* = 0.062). At 25 V, PI uptake also increased significantly at the immediate (*p* = 0.001) and 30-min (*p* = 0.008) time points; however, the magnitude of this increase was lower than that observed at 30 V. These data demonstrate a graded, voltage-dependent electroporation response, with 30 V producing the highest level of membrane permeabilization (Figure 3). 

**Figure 4:**
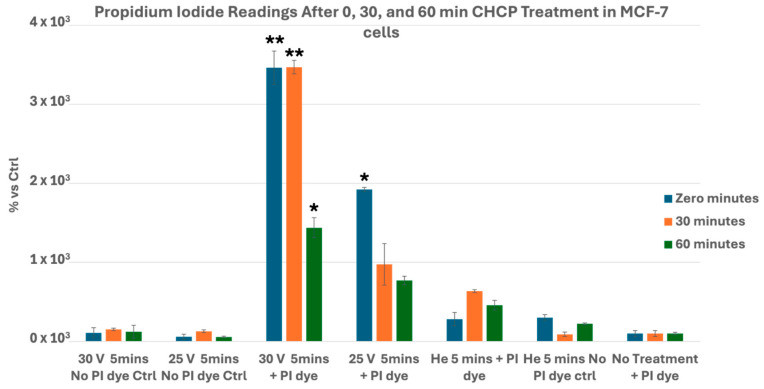
Analysis of electroporation effects of CHCP treatment on ER^+^ PR^+^ HER2^−^ breast cancer MCF-7 cells using PI intake intensity measurements. Cells were treated with CHCP at 30 V (~2010 V/cm PTEF) and 25 V for 5 min, followed by analysis at multiple incubation time points (0, 30, and 60 min) to assess the plasma-induced membrane permeability. The percentage of PI intensity indicates the degree of cell membrane electroporation, with data representing the mean ± SEM from *n* = 3 biological replicates, each containing triplicate technical measurements. Statistical significance was analyzed between CHCP-treated and helium-treated samples using Student’s *t*-test, where at 30 V, at zero, 0.5, and 1 h, it was *p* = 0.0015, *p* = 0.0089, and *p* = 0.013, respectively. At 25 V, at zero, 0.5, and 1 h, it was *p* = 0.014, *p* = 0.203, and *p* = 0.168, respectively. Data are represented by * *p* < 0.05 and ** *p* < 0.01.

In MCF-7 cells, 30 V induced robust and sustained PI uptake across all time points (*p* = 0.0015, *p* = 0.0089, *p* = 0.013), while 25 V caused a transient increase only at 0 min (*p* = 0.014), with no significant differences at 30 (*p* = 0.203) or 60 min (*p* = 0.168) (Figure 4).

**Figure 5:**
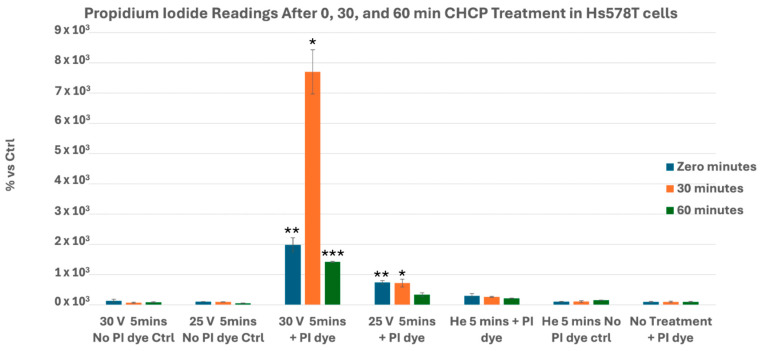
Analysis of electroporation effects of CHCP treatment on triple-negative breast cancer Hs578T cells using PI intake intensity measurements. Cells were treated with CHCP at 30 V and 25 V for 5 min, followed by analysis at multiple incubation time points (0, 30, and 60 min) to assess the plasma-induced membrane permeability. The percentage of PI intensity indicates the degree of cell membrane electroporation, with data representing the mean ± SEM from *n* = 3 biological replicates, each containing triplicate technical measurements. Statistical significance was analyzed between CHCP-treated and helium-treated samples using Student’s t-test, where at 30 V, at zero, 0.5, and 1 h, it was *p* = 0.0030, *p* = 0.0105, and *p* = 0.00014, respectively. At 25 V, at zero, 0.5, and 1 h, it was *p* = 0.0087, *p* = 0.03, and *p* = 0.783, respectively. Data are represented by * *p* < 0.05, ** *p* < 0.01 and *** *p* < 0.001.

Hs578T cells exhibited pronounced electroporation at 30 V, with significant PI uptake at 0 (*p* = 0.0030), 30 (*p* = 0.0105), and 60 min (*p* = 0.00014), whereas 25 V induced moderate early permeability at 0 (*p* = 0.0087) and 30 min (*p* = 0.03), but returned to control levels by 60 min (*p* = 0.783) (Figure 5).

**Figure 6:**
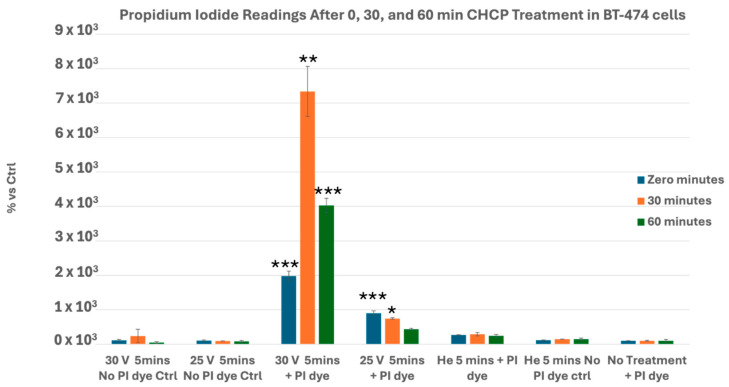
Analysis of electroporation effects of CHCP treatment on ER^+^ PR^+^ HER2^+^ breast cancer BT-474 cells using PI intake intensity measurements. Cells were treated with CHCP at 30 V and 25 V for 5 min, followed by analysis at multiple incubation time points (0, 30, and 60 min) to assess the plasma-induced membrane permeability. The percentage of PI intensity indicates the degree of cell membrane electroporation, with data representing the mean ± SEM from *n* = 3 biological replicates, each containing triplicate technical measurements. Statistical significance was analyzed between CHCP-treated and helium-treated samples using Student’s *t*-test, where at 30 V, at zero, 0.5, and 1 h, it was *p* = 0.0005, *p* = 0.0010, and *p* = 0.0001, respectively. At 25 V, at zero, 0.5, and 1 h, it was *p* = 0.0008, *p* = 0.0135, and *p* = 0.273, respectively. Data are represented by * *p* < 0.05, ** *p* < 0.01 and *** *p* < 0.001.

Similarly, BT-474 cells showed strong and sustained PI uptake at 30 V (0, 30, and 60 min; *p* = 0.0005, *p* = 0.0010, *p* = 0.0001), while 25 V elicited early and moderate increases at 0 (*p* = 0.0008) and 30 min (*p* = 0.0135), with recovery by 60 min (*p* = 0.273) (Figure 6).

To evaluate whether the observed membrane permeabilization was primarily due to the transient electric field or to plasma-generated reactive species, we conducted control experiments using plasma-activated medium (PAM) prepared at 25 V and 30 V. Three different breast cancer cell lines were incubated with PAM for 30, 60, and 120 min prior to PI staining. As shown in Supplementary Figure S4A–C, no significant increase in PI uptake was detected after 30 or 60 min of PAM incubation compared with untreated controls. A statistically significant rise in PI fluorescence was observed only after 120 min, suggesting that prolonged exposure to reactive species alone can slowly compromise membrane integrity. In contrast, cells directly exposed to CHCP under identical voltage conditions exhibited an immediate and voltage-dependent PI uptake. These findings indicate that the rapid and reversible membrane permeabilization observed during direct CHCP exposure is predominantly mediated by the electric field component, consistent with an electroporation-like mechanism.

Collectively, these data demonstrate that CHCP induces voltage- and time-dependent electroporation in breast cancer cells, with higher voltage producing robust and sustained membrane permeabilization and lower voltage eliciting transient effects. The variation in PI uptake kinetics across cell lines further indicates cell-type specific differences in membrane susceptibility. Even though the PI uptake seem to return to baseline, observed by the 60 min (*p* = 0.062) incubation in MDA-MB-231 cells, this is because of the reduction of cells in the CAP-treated samples during the cell washing process in the PI staining protocol. These results highlight the precise and reproducible nature of CHCP-induced electroporation.

### 3.2. Temperature Monitoring During CHCP Treatment

**Figure 7:**
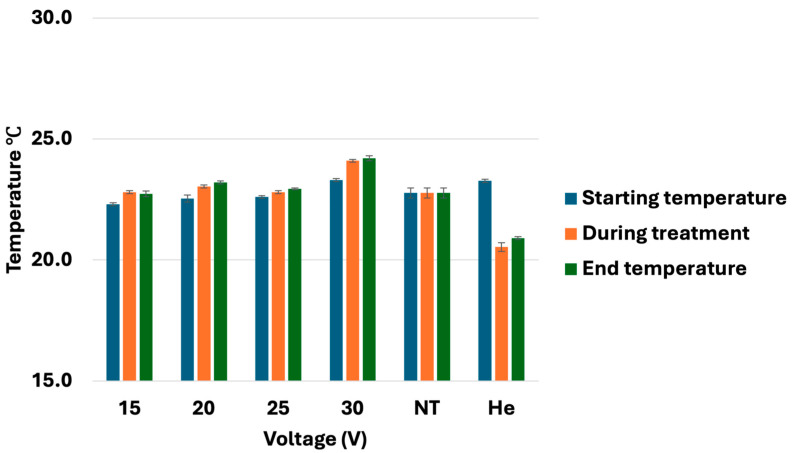
Analysis of temperature during CHCP treatment on breast cancer cells. Breast cancer cell lines MDA-MB-231, MCF-7, and Hs578T were treated with CHCP at 15 V, 20 V, 25 V, and 30 V for 5 min and temperature measurements of media were recorded before, during, and after the treatment. The figure shows the bar graphs showing the temperatures. The error bars represent means ± SEM. *(n* = 3). No Treatment (NT) and helium control (He) are at zero volts.

Temperature measurements were performed throughout CHCP treatment to assess potential thermal contributions to the observed biological effects. Across all voltages (15 V, 20 V, 25 V, and 30 V) for 5 min each, the temperature of the treated cell cultures ranged between 22 to 24 °C (Figure 7). These findings confirm that the IRE effects of CHCP, disrupting plasma membrane integrity, are attributable to a non-thermal mechanism inherent to PTEF.

### 3.3. CHCP-Induced Electroporation Facilitates Efficient BCL2A1 esiRNA Delivery and Gene Silencing

**Figure 8:**
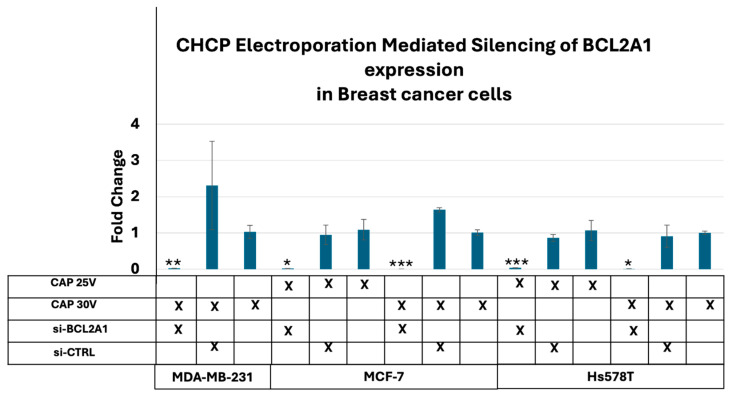
Analysis of electroporation effects of CHCP treatment on breast cancer cells by silencing of BCL2A1 mRNA. Transfection of siBCL2A1 by CHCP treatment. Breast cancer cell lines MDA-MB-231, MCF-7, and Hs578T were treated with CHCP at 15 V, 20 V, 25 V, and 30 V for 5 min. Bar graphs show the expression of BCL2A1 mRNA with esiRNA for BCL2A1 silencing with CAP treatment. The data represent the mean ± SEM from *n* = 3 biological replicates, each containing triplicate technical measurements. (MDA-MD-231 at 30 V, *p* = 0.0055; MCF-7 at 25 V, *p* = 0.012, and 30 V, *p* = 5.76253 × 10^−06^; Hs578T at 25 V, *p* = 0.0005, and 30 V, *p* = 0.02; Student’s *t*-test for siBCL2A1 versus control siRNA. Data are represented by * *p* < 0.05, ** *p* < 0.01 and *** *p* < 0.001.)

We have previously demonstrated that the anti-apoptotic gene BCL2A1 [19] is expressed following CHCP treatment. Leveraging this characteristic, we investigated the electroporation effects of CHCP by monitoring mRNA BCL2A1 expression and its silencing by esiRNA. Breast cancer cell lines MDA-MB-231, MCF-7, and Hs578T were treated with CHCP at 15 V, 20 V, 25 V, and 30 V (~2010 V/cm PTEF) for 5 min using a 3 L/min helium flow. Cells underwent CHCP treatment followed by transfection with either BCL2A1 esiRNA or control esiRNA (13 pmol) 30 min later. The total RNA was isolated after 6 h of incubation, and BCL2A1 expression was quantified by qPCR. In MDA-MB-231 cells, BCL2A1 mRNA was significantly induced by 30 V treatment and completely silenced in esiRNA-treated samples (*p* = 0.0056). In MCF-7 and Hs578T cells, BCL2A1 induction was observed at both 25 V and 30 V and was significantly silenced by esiRNA (MCF-7: 25 V, *p* = 0.013; 30 V, *p* = 5.8 × 10^−6^; Hs578T: 25 V, *p* = 4.9 × 10^−4^; 30 V, *p* = 0.02). Notably, no BCL2A1 expression was detected in any of the cell lines following 15 V or 20 V treatment. Collectively, these findings demonstrate that CHCP induces electroporation in a voltage-dependent manner, enabling BCL2A1 esiRNA to enter cells and effectively silence BCL2A1 mRNA expression (Figure 8).

### 3.4. Disruption of Plasma Membrane Integrity as a Mechanism for Inhibition of Colony Formation After Cold Plasma Treatment

**Figure 9:**
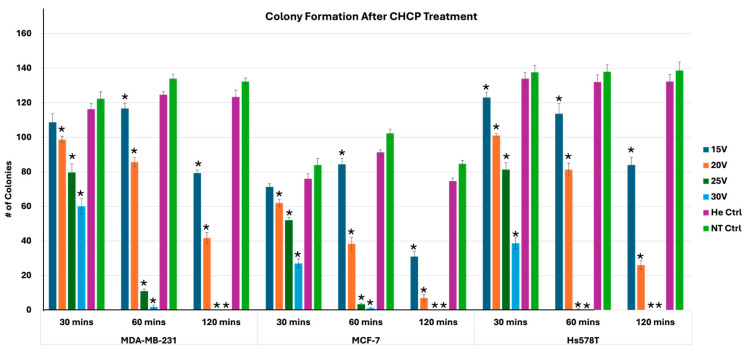
Analysis of electroporation effects of CHCP treatment on breast cancer cells on colony formation. Breast cancer cell lines MDA-MB-231, MCF-7, and Hs578T were treated with CHCP at 15 V, 20 V, 25 V, and 30 V for 5 min and incubated for 30, 60, and 120 min, followed by colony formation assays. The figure shows bar graphs showing the number of colonies with CAP treatment. The data represent the mean ± SEM from *n* = 3 biological replicates, each containing triplicate technical measurements. Significance was set at *p* < 0.05, using one-way ANOVA followed by post-hoc Tukey’s tests for treated samples versus the helium control. All *p*-values < 0 (*), indicating they were highly significant.

CHCP-induced disruption of the plasma membrane in turn leads to increased membrane permeability, uncontrolled ion fluxes, and oxidative damage to lipids and proteins, ultimately impairing the ability of cancer cells to maintain homeostasis. The downstream consequences include mitochondrial dysfunction, oxidative stress-induced signaling, DNA damage, and activation of apoptotic pathways. As the colony formation assay measures the long-term clonogenic survival of individual cells, the loss of plasma membrane integrity following cold plasma exposure directly compromises the ability of treated cancer cells to proliferate and form colonies. Thus, the observed inhibition of colony formation after cold plasma treatment reflects the combined effects of membrane destabilization, loss of viability, and failure to sustain continuous division. We investigated the electroporation effects of CHCP by examining colony formation in MDA-MB-231, MCF-7, and Hs578T breast cancer cell lines, as described in the Methods Section (Figure 9).

A comprehensive statistical analysis of colony formation was performed across these cell lines (MDA-MB-231, MCF-7, and Hs578T) treated with cold plasma at voltages ranging from 15 V to 30 V, compared to helium and non-treated controls. A total of 162 measurements (*n* = 3 per condition) were analyzed across three time points (30, 60, and 120 min). One-way ANOVA revealed highly significant treatment effects for all cell lines and time point combinations (*p* = 0.001), with F-statistics ranging from 33.3 to 742.1 (Table 2). Post-hoc Tukey’s tests confirmed significant pairwise differences between voltage-treated groups and helium controls (*p* < 0.05), indicating that inhibition of colony formation was specifically attributable to the voltage component of cold plasma exposure (Supplement Table S1A–C). Dose–response analysis demonstrated that higher voltages (25 V and 30 V) consistently produced the greatest reduction in colony formation across all cell lines, while 15 V and 20 V induced moderate but significant effects. Cell line-specific differences were observed: Hs578T cells exhibited the highest baseline clonogenic potential, followed by MDA-MB-231 and MCF-7; however, MCF-7 displayed the greatest relative sensitivity to treatment. Temporal analysis showed peak efficacy at 60 min, with significant effects sustained at 120 min, suggesting both acute and prolonged treatment responses. The inhibition of colony formation is consistent with the irreversible electroporation effect induced by CHCP treatment, which disrupts plasma membrane integrity and abolishes the ability of cancer cells to sustain long-term proliferation. Collectively, these results demonstrate robust dose- and time-dependent suppression of clonogenic survival across multiple breast cancer cell lines, validating the potential of irreversible electroporation mediated by CHCP.

### 3.5. CHCP Induced Morphological Changes Consistent with Irreversible Electroporation

Phase contrast microscopy images (Supplemental Figures S1–S3) revealed distinct morphological changes in breast cancer cells following CHCP treatment at 25 V and 30 V. In untreated Hs578T (ER^−^/PR^−^/HER2^−^) cells, membranes exhibited well-defined borders (black arrows), whereas treated cells displayed membrane blebbing and cell shrinkage within 1 h, indicative of oxidative stress (red arrows). Cells exposed to 25 V showed no recovery in morphology and detached from the plate by 24 h post-treatment, while those treated at 30 V detached as early as 6 h. Similar morphological patterns were observed in BT-474 (ER^+^/PR^+^/HER2^+^) cells, where CHCP treatment induced membrane blebbing and shrinkage by 1 h and subsequent detachment at 24 h (25 V) and 6 h (30 V). In MDA-MB-231 (ER^−^/PR^−^/HER2^−^) cells, blebbing and shrinkage were evident by 1 h, with detachment occurring at 6 h (25 V) and 4 h (30 V). No such changes were observed in the non-treated (NT) or helium (He) control groups, confirming that the effects were specific to the voltage component of CHCP exposure. The progressive membrane blebbing, shrinkage, and detachment are consistent with irreversible electroporation and oxidative stress-mediated damage, which compromise plasma membrane integrity in a voltage- and dose-dependent manner. These results demonstrate that CHCP disrupts cellular homeostasis through non-thermal electroporation effects, ultimately leading to loss of viability and detachment in multiple breast cancer cell lines.

### 3.6. Ex Vivo Histopathology Reveals Selective Tumor Cell Death via CHCP-Induced Irreversible Electroporation in Human Solid Tumors

**Figure 10:**
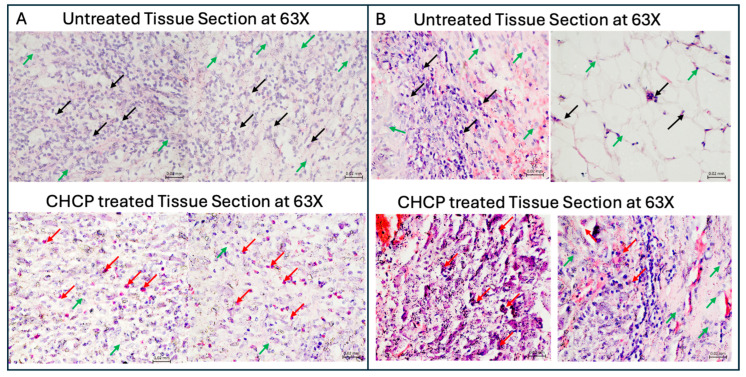
Light micrographs of H&E-stained Zone 0 tissue samples from patient (**A**) R0009 (metastatic pleomorphic sarcoma of the left distal femur) and (**B**) R0004 (metastatic recurrent non-small cell lung adenocarcinoma of the left hip/proximal femur) with or without CHCP treatment, imaged at 63× objective magnification. Black arrows indicate viable untreated tumor cells, red arrows denote CHCP-treated tumor cells exhibiting apoptosis with cellular cytosolic content leakage, and green arrows represent normal cells. Scale bars = 0.2 mm.

We re-examined and analyzed formalin-fixed paraffin-embedded (FFPE) tissue sections obtained from stage IV or recurrent solid tumors in patients enrolled in our recent Phase I clinical study (NCT04267575) [14]. The baseline demographic and clinical characteristics of participants in each study condition were also detailed in our previous study [14]. Among the 12 patients included, 60% achieved R0 resection status. Tissue specimens were collected at the surgical margins (Zone 0), and resected samples were subjected to ex vivo CHCP treatment to evaluate the localized effects on tumor and adjacent non-tumor tissue. In this study, two FFPE patients’ sample sections were used, as mentioned in the Methods Section. Histopathological evaluation using hematoxylin and eosin (H&E) staining revealed selective CHCP-induced tumor cell death in the resected samples (Figure 10A,B). Images are taken from a dataset of *n* = 5 R0-resected patient specimens analyzed for post-treatment cellular and structural alterations. The micrographs illustrate distinct differences between treated and untreated regions, showing selective tumor cell detachment and morphological disruption consistent with CHCP-induced non-thermal electroporation effects. These images are presented as illustrative examples of the broader dataset. Morphological changes included compromised plasma membrane integrity and cellular swelling, culminating in the leakage of cellular contents. These features are consistent with an IRE mechanism, as observed in our in vitro studies where CHCP produced voltage-dependent membrane permeabilization across breast cancer subtypes. Importantly, CHCP-induced cytotoxic effects were confined to malignant cells within the treated margins, while adjacent structures, including connective tissue and vascular components, remained largely preserved. There was no widespread necrosis or nonspecific collateral damage observed in the surrounding non-tumor regions. The results provide translational evidence that CHCP selectively targets tumor cells via NTNC-IRE mechanisms in both preclinical models and clinical specimens.

## 4. Discussion

Irreversible electroporation (IRE) has been investigated for over two decades as a non-thermal tumor ablation strategy, with foundational work by Robinsky [20,21] demonstrating that externally applied electric fields can disrupt plasma membrane integrity through nanopore formation. Conventional clinical systems such as NanoKnife [22] and Galvanize Knife [23] deliver high-voltage pulses via direct electrode insertion, creating transient pores that lead to cell death. While effective, these platforms are invasive, may introduce thermal artifacts, and often lack direct evidence of strictly non-thermal electroporation [2,3,4,24].

In contrast, the Canady Helios Cold Plasma (CHCP) system induces membrane permeabilization through a non-contact, non-thermal combination of transient electromagnetic fields and plasma-generated species. Our in vitro data demonstrate that CHCP produces clearly voltage- and time-dependent electroporation across multiple breast cancer subtypes. At a higher PTEF (~2010 V/cm, 30 V), MDA-MB-231, MCF-7, Hs578T, and BT-474 cells exhibited robust, sustained PI uptake, whereas intermediate PTEF (~1675 V/cm, 25 V) yielded only transient permeability that diminished within 30–60 min. Cell-type differences were apparent: triple-negative Hs578T and HER2-positive BT-474 retained elevated PI uptake at 30 V longer than hormone-receptor-positive MCF-7 cells.

Variations in PI uptake kinetics reflect intrinsic differences in membrane lipid composition, cytoskeletal stiffness, and repair capacity [25]. In MDA-MB-231 and BT-474 cells, PI uptake continued to increase during the first 30 min following CHCP exposure. This is consistent with established electroporation models in which nascent nanopores may transiently expand or stabilize even after the external field is removed, allowing continued dye influx. CHCP-induced oxidative membrane weakening may further prolong permeability in some lines. Importantly, the subsequent decrease in PI fluorescence observed at 60 min across all cell lines does not indicate pore closure or recovery. Instead, this reduction is attributable to the loss of CHCP-compromised cells during the washing steps required for PI staining, decreasing the total number of cells available for imaging and thereby reducing the overall PI signal. This methodological limitation is now acknowledged. Given intrinsic differences in lipid content [26] and baseline membrane fluidity among MDA-MB-231, MCF-7, and BT-474 cells, variation in pore formation thresholds and post-treatment cell adherence likely contribute to the heterogeneous PI uptake patterns observed here.

PAM experiments provided critical mechanistic separation. Because PAM contains long-lived RONS but no electric field, PAM exposure produced no PI uptake within 60 min and only delayed permeability at 120 min, indicating that oxidative injury alone acts on a slower timescale. In contrast, direct CHCP exposure induced immediate, voltage-dependent PI uptake, confirming that the transient electric field, not RONS alone, is responsible for rapid membrane disruption. Although a grounded-grid shield could theoretically further isolate electric-field effects, such structures fundamentally alter plasma jet behavior; plume stability in CHCP is highly sensitive to the axial position of the powered electrode and nearby grounded surfaces, and even millimeter-scale changes can shift the jet from a stable guided plume to a weak or unstable discharge [27]. A grid would therefore modify breakdown physics, power coupling, electron density, and the generation of short-lived species, creating a non-representative exposure condition. For this reason, PAM serves as the appropriate mechanistic control, preserving long-lived RONS while removing both the electric field and plume-confined short-lived species. The absence of rapid PI uptake under PAM strongly supports an electric-field-dominant mechanism, although we acknowledge that short-lived species at the plasma liquid interface cannot yet be fully decoupled from the PTEF in the current system.

Recent mechanistic work in plasma medicine reinforces our interpretation that rapid CAP-induced membrane permeabilization is driven primarily by electric-field-associated short-lived species rather than long-lived oxidants [28]. Studies using 3D hydrogels, tissue-like matrices, and ex vivo models show that gas plasma-derived long-lived species (e.g., H_2_O_2_, NO_2_^−^, and NO_3_^−^) penetrate tissue poorly and are rapidly consumed by biomolecules, whereas short-lived ROS such as •OH, O, and ^1^O_2_ drive most oxidative modifications [28]. Direct plasma exposure consistently produces much stronger cytotoxicity than PAM, underscoring the limited biological impact of long-lived species alone. Furthermore, catalase-embedded tissue models show no rescue effect, confirming the negligible contribution of H_2_O_2_ in tissue-like environments and highlighting the predominance of short-lived species in plasma–tissue interactions [28]. These insights align with our findings that PAM fails to induce rapid PI uptake, whereas direct CHCP generates immediate, voltage-dependent electroporation.

Morphological changes, including membrane blebbing, shrinkage, and detachment, corroborated the electroporation mechanism. Cells exposed to 30 V detached within 4–6 h, whereas 25 V required ~24 h, consistent with the increasing severity of irreversible membrane damage. Untreated and helium controls showed no such alterations.

Functionally, CHCP-generated pores enabled intracellular macromolecule transport. BCL2A1-targeting esiRNA was delivered only at higher PTEF values (~1675–2010 V/cm), establishing a voltage threshold for stable pore formation. Gene silencing and suppressed clonogenic survival confirmed effective intracellular access. These results align with studies showing CAP systems can deliver plasmids, DNA, siRNA, and miRNA in vitro and in vivo [15], and with microsecond-pulsed DBD research demonstrating CAP-induced poration that reduces TEER in a voltage-dependent manner [29].

The present findings fit within known electroporation biophysics. CAP-generated electric fields can create localized transmembrane potentials sufficient to exceed dielectric thresholds (~1–1.5 V/nm), forming transient pores [30,31,32]. RONS then amplify downstream damage through lipid peroxidation, protein oxidation, mitochondrial dysfunction, and DNA injury [33,34]. Tumor cells are particularly susceptible due to disordered lipid organization, depolarized resting potentials, elevated oxidative stress, and impaired membrane repair [16,35,36,37]. Together, PTEF and RONS form a dual-action mechanism: rapid electric-field-mediated poration followed by slower oxidative injury.

This mechanistic framework aligns with immunogenic cell death pathways. Irreversible membrane damage promotes the release of calreticulin, ATP, HMGB1, and other DAMPs, which activate antigen-presenting cells and prime adaptive immune responses [38,39]. Thus, CHCP-induced electroporation may contribute not only to immediate cytotoxicity but also to antitumor immunomodulation.

Clinical relevance was supported by ex vivo analyses of Zone 0 tissues from our Phase I trial (NCT04267575) [1]. H&E staining revealed selective tumor cell injury characterized by membrane disruption, cytoplasmic leakage, and nuclear condensation, while adjacent non-malignant tissues remained preserved. These observations parallel our in vitro findings and suggest that CHCP can target residual tumor cells at the surgical margin without collateral thermal injury. Patients with microscopic residual disease who received CHCP application exhibited improved postoperative outcomes, indicating the potential clinical benefit of intraoperative CHCP treatment.

The ability to induce non-thermal electroporation without physical contact has important implications for surgical oncology [40,41,42]. Non-contact permeabilization may permit treatment of irregular or otherwise inaccessible post-resection margins, where residual tumor cells can persist despite clear gross margins. CHCP offers several advantages over conventional IRE platforms: non-contact operation, preserved non-thermal conditions (22–24 °C), precise voltage control, and cell-type-dependent selectivity. By integrating controlled electric-field effects with plasma-generated RONS, CHCP functions as a novel NTNC-IRE platform capable of inducing both immediate electroporation and sustained tumor-selective cytotoxicity. Our study suggest that plasma-based NTNC-IRE may serve as an adjunctive selective margin–tumor cell death technology, warranting further clinical evaluation.

**Figure 11:**
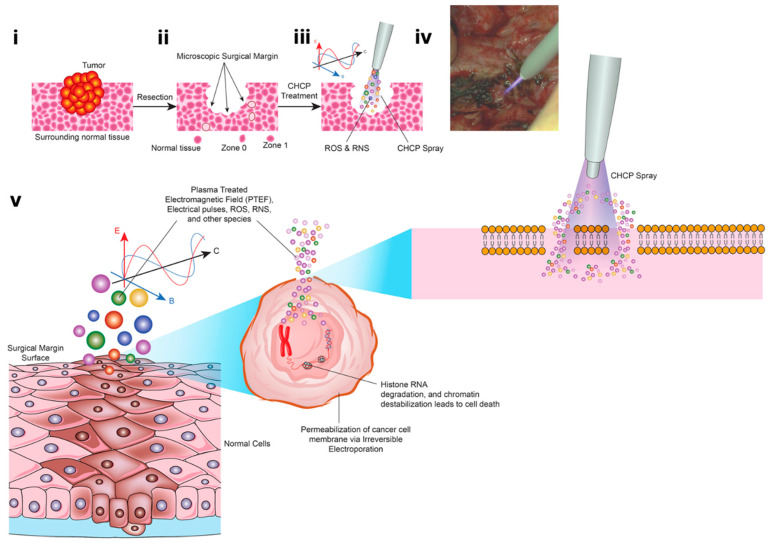
Schematic representation of the Canady Helios Cold Plasma (CHCP) treatment workflow. The illustration depicts (**i**) surgical resection of the tumor using standard oncologic procedures, (**ii**) collection of specimens from the tumor core, surgical margin (Zone 0), peritumoral tissue (Zone 1), and adjacent normal tissue, and (**iii**) intraoperative application of the CHCP spray directly to the surgical margin. (**iv**) A representative intraoperative image shows CHCP treatment of a pancreatic surgical margin following a Whipple procedure. (**v**) The schematic further highlights the CHCP-generated PTEF, where E denotes the electric field, B the magnetic field, and C the three-dimensional vector reference of the magnetic field. Reactive oxygen and nitrogen species (ROS/RNS), including hydroxyl radicals, are shown entering the cancer cell membrane through transient “pores” formed by CHCP-induced electroporation, ultimately disrupting cellular integrity and promoting selective tumor cell death.

Overall, this study demonstrates that CHCP induces voltage-dependent, non-contact electroporation that enables functional siRNA delivery, promotes tumor-selective membrane disruption, and supports margin-focused cancer control. These mechanistic insights provide a strong foundation for clinical translation of CHCP as an intraoperative adjunct to reduce residual tumor burden and improve local recurrence outcomes in solid tumors (Figure 11).

## 5. Conclusions

The CHCP system is the first next-generation, non-thermal (22–24 °C), non-contact electroporation biophysical platform that induces selective tumor cell death through NTNC-IRE. CHCP integrates plasma-generated reactive species with a PTEF to kill malignant cells while preserving normal tissue architecture. Clinically, this approach enables an intraoperative strategy which may prevent local recurrence, potentially improving patient outcomes and survival.

## Figures and Tables

**Table 1:** Table listing PTEF Field Strength (V/cm) corresponding to input voltage (V) used in this study.

Input Setting Voltage (V)	PTEF Field Strength (V/cm)
15 V	~1005 V/cm
20 V	~1340 V/cm
25 V	~1675 V/cm
30 V	~2010 V/cm

**Table 2:** ANOVA results for colony formation across cell lines and time points.

Cell Line	Time Point	F-Statistic	*p*-Value
MDA-MB-231	30 min	33.3	1.23 × 10^−6^
MDA-MB-231	60 min	742.1	1.64 × 10^−14^
MDA-MB-231	120 min	626.3	4.52 × 10^−14^
MCF-7	30 min	63.1	3.38 × 10^−8^
MCF-7	60 min	353.2	1.37 × 10^−12^
MCF-7	120 min	410.7	5.6 × 10^−13^
Hs578T	30 min	131.1	4.84 × 10^−10^
Hs578T	60 min	289.1	4.52 × 10^−12^
Hs578T	120 min	358.4	1.26 × 10^−12^

## Data Availability

The original contributions presented in this study are included in the article/Supplementary Material. Further inquiries can be directed to the corresponding author.

## Supplementary Materials

The following supporting information can be downloaded at: https://www.mdpi.com/article/10.3390/cancers17233869/s1, Supplemental Figure S1: Phase contrast microscopy images showing voltage-dependent morphological disruption of Hs578T cells following CHCP treatment; Figure S2: Phase contrast microscopy images voltage-dependent morphological disruption of BT-474 cells following CHCP treatment; Figure S3: Phase contrast microscopy images voltage-dependent morphological disruption of MDA-MB-231 cells following CHCP treatment; and Figure S4A: Analysis of Electroporation effects of PAM on triple-negative breast cancer MDA-MB-231 cells using PI intake intensity measurements; Figure S4B: Analysis of Electroporation effects of PAM on triple-negative breast cancer MCF-7 cells using PI intake intensity measurements; Figure S4C: Analysis of Electroporation effects of PAM on triple-negative breast cancer BT-474 cells using PI intake intensity measurements; Table S1A: Tukey’s HSD Post Hoc Test Results for MDA-MB-231 Cell Line colony formation assay; Table S1B: Tukey’s HSD Post Hoc Test Results for MCF-7 Cell Line colony formation assay; and Table S1C: Tukey’s HSD Post Hoc Test Results for Hs578T Cell Line colony formation assay.
