# Supplementary material for: Canady Helios Cold Plasma Induces Non-Thermal (24 °C), Non-Contact Irreversible Electroporation and Selective Tumor Cell Death at Surgical Margins
Source: Cancers (Basel). 2025 Dec 2;17(23):3869. doi: 10.3390/cancers17233869 (PMC12691019; doi:10.3390/cancers17233869)
Supplement: Supplementary file 1 [file cancers-17-03869-s001.zip › Supplement Table S1.pdf]

Supplement Table S1A:
Table S1A. Tukey's HSD Post Hoc Test Results for MDA-MB-231 Cell Line

Time Point	Comparison	Mean Diff	p-adj	Sig
30 min	15V vs 20V	-10	0.55	ns
30 min	15V vs 25V	-29	0	**
30 min	15V vs 30V	-48.67	<0.000001	
30 min	15V vs Helium control	7.67	0.77	ns
30 min	15V vs NT control	13.67	0.25	ns
30 min	20V vs 25V	-19	0.06	ns
30 min	20V vs 30V	-38.67	0	
30 min	20V vs Helium control	17.67	0.09	ns
30 min	20V vs NT control	23.67	0.02	*
30 min	25V vs 30V	-19.67	0.05	*
30 min	25V vs Helium control	36.67	0	
30 min	25V vs NT control	42.67	0	
30 min	30V vs Helium control	56.33	<0.000001	
30 min	30V vs NT control	62.33	<0.000001	
30 min	Helium control vs NT control	6	0.9	ns
**60 min**	**15V vs 20V**	**-31**	**<0.000001**	*******
**60 min**	**15V vs 25V**	**-105.67**	**<0.000001**	*******
**60 min**	**15V vs 30V**	**-115**	**<0.000001**	*******
60 min	15V vs Helium control	8	0.16	ns
60 min	15V vs NT control	17.33	0	**
**60 min**	**20V vs 25V**	**-74.67**	**<0.000001**	*******

**60 min**	**20V vs 30V**	**-84**	**<0.000001**	*******
**60 min**	**20V vs Helium control**	**39**	**<0.000001**	*******
**60 min**	**20V vs NT control**	**48.33**	**<0.000001**	*******
60 min	25V vs 30V	-9.33	0.08	ns
**60 min**	**25V vs Helium control**	**113.67**	**<0.000001**	*******
**60 min**	**25V vs NT control**	**123**	**<0.000001**	*******
**60 min**	**30V vs Helium control**	**123**	**<0.000001**	*******
**60 min**	**30V vs NT control**	**132.33**	**<0.000001**	*******
60 min	Helium control vs NT control	9.33	0.08	ns
**120 min**	**15V vs 20V**	**-37.67**	**<0.000001**	*******
**120 min**	**15V vs 25V**	**-79.33**	**<0.000001**	*******
**120 min**	**15V vs 30V**	**-79.33**	**<0.000001**	*******
**120 min**	**15V vs Helium control**	**44**	**<0.000001**	*******
**120 min**	**15V vs NT control**	**53**	**<0.000001**	*******
**120 min**	**20V vs 25V**	**-41.67**	**<0.000001**	*******
**120 min**	**20V vs 30V**	**-41.67**	**<0.000001**	*******
**120 min**	**20V vs Helium control**	**81.67**	**<0.000001**	*******
**120 min**	**20V vs NT control**	**90.67**	**<0.000001**	*******
120 min	25V vs 30V	0	1	ns
**120 min**	**25V vs Helium control**	**123.33**	**<0.000001**	*******
**120 min**	**25V vs NT control**	**132.33**	**<0.000001**	*******
**120 min**	**30V vs Helium control**	**123.33**	**<0.000001**	*******
**120 min**	**30V vs NT control**	**132.33**	**<0.000001**	*******
120 min	Helium control vs NT control	9	0.14	ns

Supplement Table S1B:
Table S1B. Tukey's HSD Post Hoc Test Results for MCF-7 Cell Line

**Time Point**	**Comparison**	**Mean Diff**	**p-adj**	**Sig**
30 min	15V vs 20V	-9.33	0.18	ns
30 min	15V vs 25V	-19.33	0	**
30 min	15V vs 30V	-44.33	<0.000001	
30 min	15V vs Helium control	4.67	0.79	ns
30 min	15V vs NT control	12.67	0.04	*

30 min	20V vs 25V	-10	0.14	ns
30 min	20V vs 30V	-35	<0.000001	
30 min	20V vs Helium control	14	0.02	*
30 min	20V vs NT control	22	0	
30 min	25V vs 30V	-25	0	
30 min	25V vs Helium control	24	0	
30 min	25V vs NT control	32	<0.000001	
30 min	30V vs Helium control	49	<0.000001	
30 min	30V vs NT control	57	<0.000001	
30 min	Helium control vs NT control	8	0.31	ns
**60 min**	**15V vs 20V**	**-46**	**<0.000001**	*******
**60 min**	**15V vs 25V**	**-81**	**<0.000001**	*******
**60 min**	**15V vs 30V**	**-83.33**	**<0.000001**	*******
60 min	15V vs Helium control	7	0.37	ns
60 min	15V vs NT control	18	0	**
**60 min**	**20V vs 25V**	**-35**	**<0.000001**	*******
**60 min**	**20V vs 30V**	**-37.33**	**<0.000001**	*******
**60 min**	**20V vs Helium control**	**53**	**<0.000001**	*******
**60 min**	**20V vs NT control**	**64**	**<0.000001**	*******
60 min	25V vs 30V	-2.33	0.98	ns
**60 min**	**25V vs Helium control**	**88**	**<0.000001**	*******
**60 min**	**25V vs NT control**	**99**	**<0.000001**	*******
**60 min**	**30V vs Helium control**	**90.33**	**<0.000001**	*******
**60 min**	**30V vs NT control**	**101.33**	**<0.000001**	*******

60 min	Helium control vs NT control	11	0.06	ns
120 min	15V vs 20V	-24	<0.000001	***
120 min	15V vs 25V	-31	<0.000001	***
120 min	15V vs 30V	-31	<0.000001	***
120 min	15V vs Helium control	43.67	<0.000001	***
120 min	15V vs NT control	53.67	<0.000001	***
120 min	20V vs 25V	-7	0.16	ns
120 min	20V vs 30V	-7	0.16	ns
120 min	20V vs Helium control	67.67	<0.000001	***
120 min	20V vs NT control	77.67	<0.000001	***
120 min	25V vs 30V	0	1	ns
120 min	25V vs Helium control	74.67	<0.000001	***
120 min	25V vs NT control	84.67	<0.000001	***
120 min	30V vs Helium control	74.67	<0.000001	***
120 min	30V vs NT control	84.67	<0.000001	***
120 min	Helium control vs NT control	10	0.03	*

Supplement Table S1C:
Table S1C. Tukey's HSD Post Hoc Test Results for Hs578T Cell Line

Time Point	Comparison	Mean Diff	p-adj	Sig
30 min	15V vs 20V	-22	0.01	**
30 min	15V vs 25V	-41.67	<0.000001	
30 min	15V vs 30V	-84.33	<0.000001	
30 min	15V vs Helium control	11	0.25	ns
30 min	15V vs NT control	14.67	0.07	ns
30 min	20V vs 25V	-19.67	0.01	*
30 min	20V vs 30V	-62.33	<0.000001	
30 min	20V vs Helium control	33	0	

**30 min**	**20V vs NT control**	**36.67**	**0**	
**30 min**	**25V vs 30V**	**-42.67**	**<0.000001**	
**30 min**	**25V vs Helium control**	**52.67**	**<0.000001**	
**30 min**	**25V vs NT control**	**56.33**	**<0.000001**	
**30 min**	**30V vs Helium control**	**95.33**	**<0.000001**	
**30 min**	**30V vs NT control**	**99**	**<0.000001**	
**30 min**	**Helium control vs NT control**	**3.67**	**0.97**	**ns**
**60 min**	**15V vs 20V**	**-32.33**	**0.0005**	*****
**60 min**	**15V vs 25V**	**-113.33**	**<0.000001**	*****
**60 min**	**15V vs 30V**	**-113.67**	**<0.000001**	*****
**60 min**	**15V vs Helium control**	**18.33**	**0.04**	*
**60 min**	**15V vs NT control**	**24.33**	**0.01**	**
**60 min**	**20V vs 25V**	**-81**	**<0.000001**	*****
**60 min**	**20V vs 30V**	**-81.33**	**<0.000001**	*****
**60 min**	**20V vs Helium control**	**50.67**	**<0.000001**	*****
**60 min**	**20V vs NT control**	**56.67**	**<0.000001**	*****
**60 min**	**25V vs 30V**	**-0.33**	**1**	**ns**
**60 min**	**25V vs Helium control**	**131.67**	**<0.000001**	*****
**60 min**	**25V vs NT control**	**137.67**	**<0.000001**	*****
**60 min**	**30V vs Helium control**	**132**	**<0.000001**	*****
**60 min**	**30V vs NT control**	**138**	**<0.000001**	*****
**60 min**	**Helium control vs NT control**	**6**	**0.85**	**ns**
**120 min**	**15V vs 20V**	**-58**	**<0.000001**	*****
**120 min**	**15V vs 25V**	**-84**	**<0.000001**	*****

**120 min**	**15V vs 30V**	**-84**	**<0.000001**	*******
**120 min**	**15V vs Helium control**	**48.33**	**<0.000001**	*******
**120 min**	**15V vs NT control**	**54.67**	**<0.000001**	*******
**120 min**	**20V vs 25V**	**-26**	**0**	******
**120 min**	**20V vs 30V**	**-26**	**0**	******
**120 min**	**20V vs Helium control**	**106.33**	**<0.000001**	*******
**120 min**	**20V vs NT control**	**112.67**	**<0.000001**	*******
**120 min**	**25V vs 30V**	**0**	**1**	**ns**
**120 min**	**25V vs Helium control**	**132.33**	**<0.000001**	*******
**120 min**	**25V vs NT control**	**138.67**	**<0.000001**	*******
**120 min**	**30V vs Helium control**	**132.33**	**<0.000001**	*******
**120 min**	**30V vs NT control**	**138.67**	**<0.000001**	*******
**120 min**	**Helium control vs NT control**	**6.33**	**0.76**	**ns**

Legend:

*** p < 0 (highly significant)
** p < 0.01 (very significant)
* p < 0.05 (significant)
ns p ≥ 0.05 (not significant)

Mean Diff: Difference in mean colony count between groups
p-adj: p-value adjusted for multiple comparisons using Tukey's HSD method
Sig: Significance level

Summary Statistics:

Total comparisons: 135 (15 comparisons × 3 cell lines × 3 time points)
Significant comparisons: 107/135 (79.3%)
Multiple comparison correction: Tukey's HSD method (α = 0.05)

These comprehensive tables provide all pairwise comparison results for your scientific publication, showing the robust statistical evidence for treatment effects across all experimental conditions.
