# Supplementary material for: Canady Helios Cold Plasma Induces Non-Thermal (24 °C), Non-Contact Irreversible Electroporation and Selective Tumor Cell Death at Surgical Margins
Source: Cancers (Basel). 2025 Dec 2;17(23):3869. doi: 10.3390/cancers17233869 (PMC12691019; doi:10.3390/cancers17233869)
Supplement: Supplementary file 1 [file cancers-17-03869-s001.zip › Supplemental Figure S1.pptx]

## Slide 1
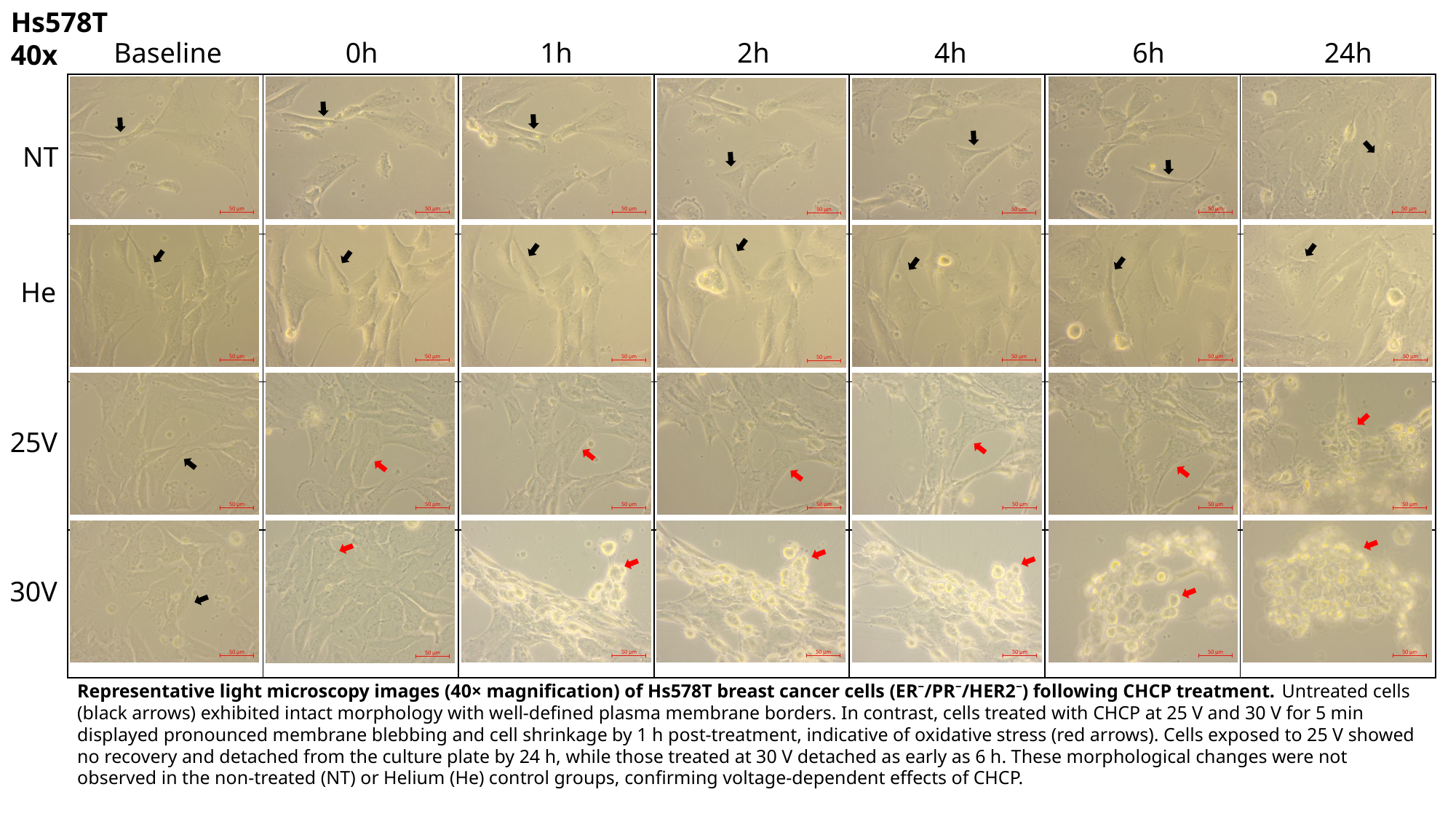

Hs578T
40x
Baseline
0h
1h
2h
4h
6h
24h
| | | | | | | |
| --- | --- | --- | --- | --- | --- | --- |
| | | | | | | |
| | | | | | | |
| | | | | | | |
NT
He
25V
30V
Representative light microscopy images (40× magnification) of Hs578T breast cancer cells (ER⁻/PR⁻/HER2⁻) following CHCP treatment. Untreated cells (black arrows) exhibited intact morphology with well-defined plasma membrane borders. In contrast, cells treated with CHCP at 25 V and 30 V for 5 min displayed pronounced membrane blebbing and cell shrinkage by 1 h post-treatment, indicative of oxidative stress (red arrows). Cells exposed to 25 V showed no recovery and detached from the culture plate by 24 h, while those treated at 30 V detached as early as 6 h. These morphological changes were not observed in the non-treated (NT) or Helium (He) control groups, confirming voltage-dependent effects of CHCP.
