# Supplementary material for: Canady Helios Cold Plasma Induces Non-Thermal (24 °C), Non-Contact Irreversible Electroporation and Selective Tumor Cell Death at Surgical Margins
Source: Cancers (Basel). 2025 Dec 2;17(23):3869. doi: 10.3390/cancers17233869 (PMC12691019; doi:10.3390/cancers17233869)
Supplement: Supplementary file 1 [file cancers-17-03869-s001.zip › Supplemental Figure S2.pptx]

## Slide 1
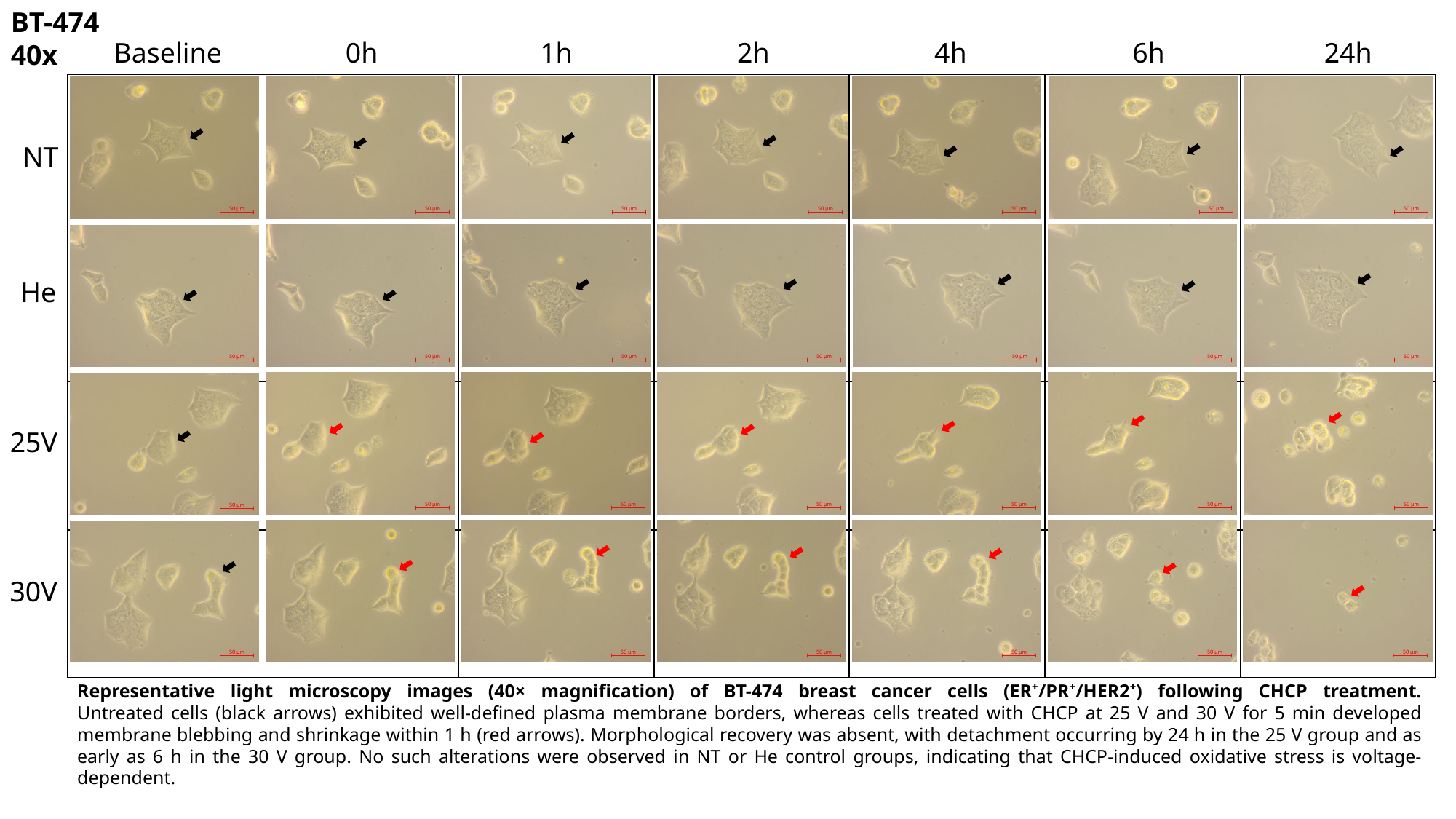

BT-474
40x
Baseline
0h
1h
2h
4h
6h
24h
| | | | | | | |
| --- | --- | --- | --- | --- | --- | --- |
| | | | | | | |
| | | | | | | |
| | | | | | | |
NT
He
25V
30V
Representative light microscopy images (40× magnification) of BT-474 breast cancer cells (ER⁺/PR⁺/HER2⁺) following CHCP treatment.
Untreated cells (black arrows) exhibited well-defined plasma membrane borders, whereas cells treated with CHCP at 25 V and 30 V for 5 min developed membrane blebbing and shrinkage within 1 h (red arrows). Morphological recovery was absent, with detachment occurring by 24 h in the 25 V group and as early as 6 h in the 30 V group. No such alterations were observed in NT or He control groups, indicating that CHCP-induced oxidative stress is voltage-dependent.
