# Supplementary material for: Canady Helios Cold Plasma Induces Non-Thermal (24 °C), Non-Contact Irreversible Electroporation and Selective Tumor Cell Death at Surgical Margins
Source: Cancers (Basel). 2025 Dec 2;17(23):3869. doi: 10.3390/cancers17233869 (PMC12691019; doi:10.3390/cancers17233869)
Supplement: Supplementary file 1 [file cancers-17-03869-s001.zip › Supplemental Figure S3.pptx]

## Slide 1
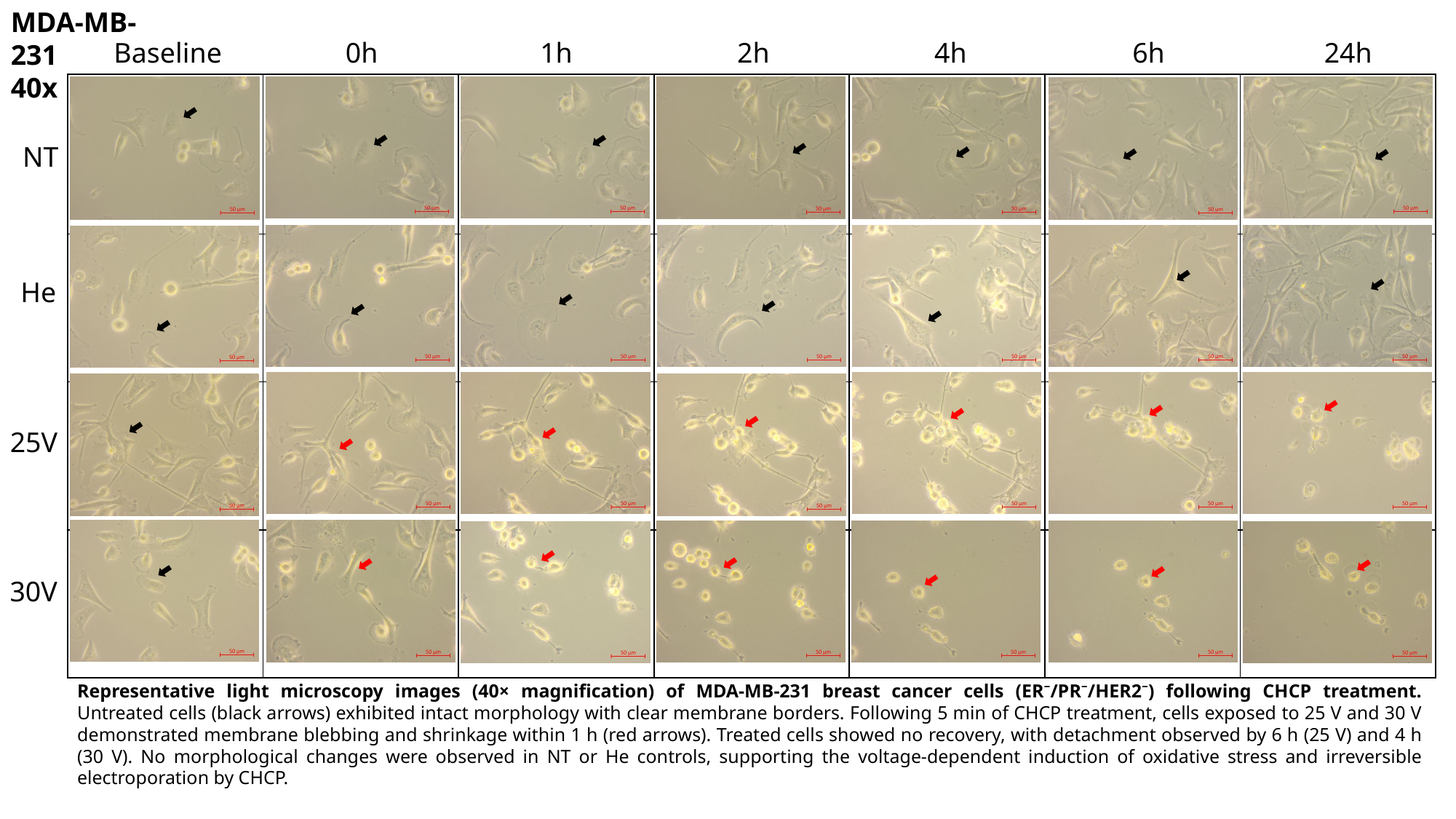

MDA-MB-231
40x
Baseline
0h
1h
2h
4h
6h
24h
| | | | | | | |
| --- | --- | --- | --- | --- | --- | --- |
| | | | | | | |
| | | | | | | |
| | | | | | | |
NT
He
25V
30V
Representative light microscopy images (40× magnification) of MDA-MB-231 breast cancer cells (ER⁻/PR⁻/HER2⁻) following CHCP treatment.
Untreated cells (black arrows) exhibited intact morphology with clear membrane borders. Following 5 min of CHCP treatment, cells exposed to 25 V and 30 V demonstrated membrane blebbing and shrinkage within 1 h (red arrows). Treated cells showed no recovery, with detachment observed by 6 h (25 V) and 4 h (30 V). No morphological changes were observed in NT or He controls, supporting the voltage-dependent induction of oxidative stress and irreversible electroporation by CHCP.
