# Supplementary material for: Canady Helios Cold Plasma Induces Non-Thermal (24 °C), Non-Contact Irreversible Electroporation and Selective Tumor Cell Death at Surgical Margins
Source: Cancers (Basel). 2025 Dec 2;17(23):3869. doi: 10.3390/cancers17233869 (PMC12691019; doi:10.3390/cancers17233869)
Supplement: Supplementary file 1 [file cancers-17-03869-s001.zip › Supplemental Figure S4.pptx]

## Slide 1
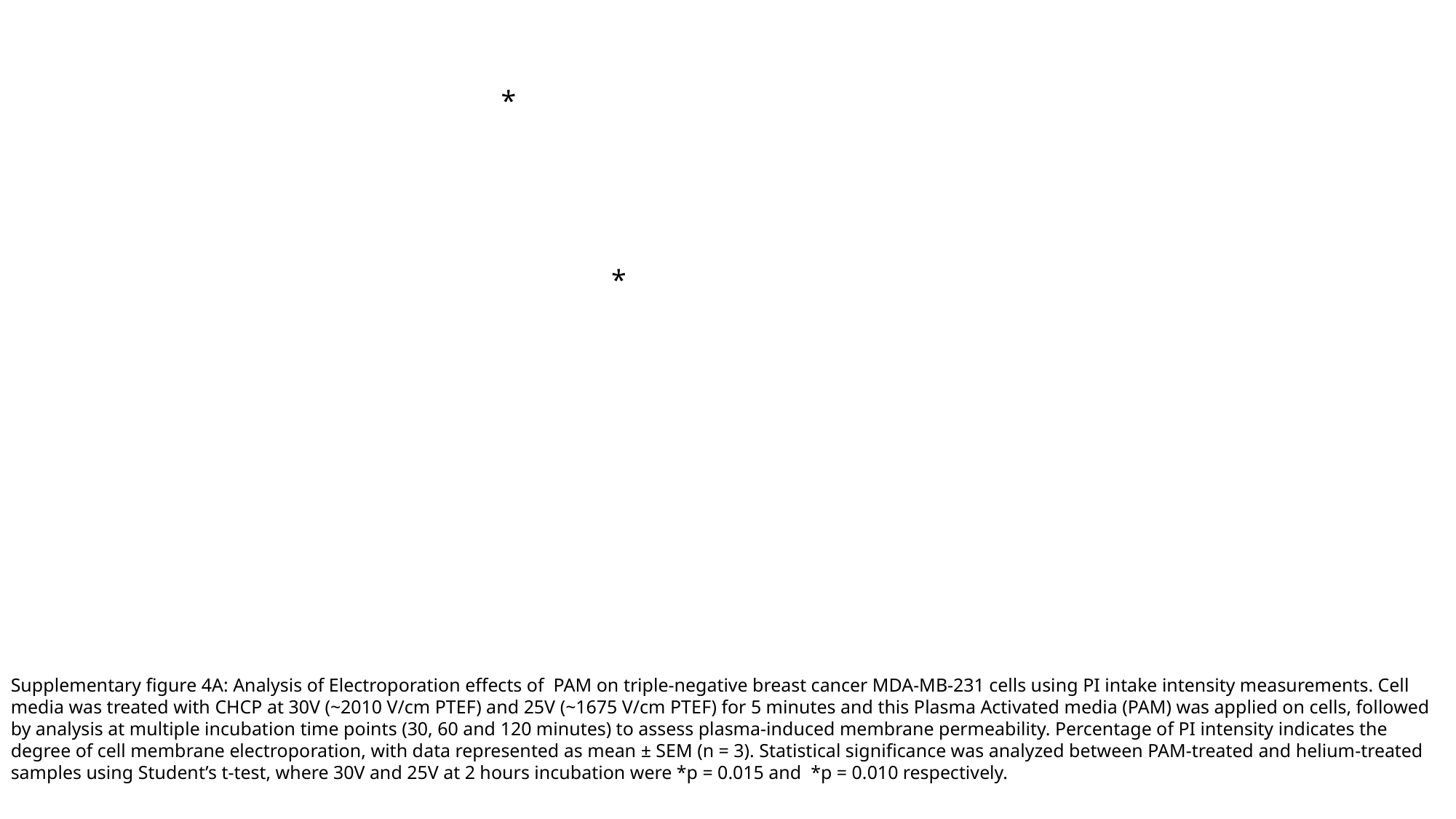

*
*
Supplementary figure 4A: Analysis of Electroporation effects of PAM on triple-negative breast cancer MDA-MB-231 cells using PI intake intensity measurements. Cell media was treated with CHCP at 30V (~2010 V/cm PTEF) and 25V (~1675 V/cm PTEF) for 5 minutes and this Plasma Activated media (PAM) was applied on cells, followed by analysis at multiple incubation time points (30, 60 and 120 minutes) to assess plasma-induced membrane permeability. Percentage of PI intensity indicates the degree of cell membrane electroporation, with data represented as mean ± SEM (n = 3). Statistical significance was analyzed between PAM-treated and helium-treated samples using Student’s t-test, where 30V and 25V at 2 hours incubation were *p = 0.015 and *p = 0.010 respectively.

## Slide 2
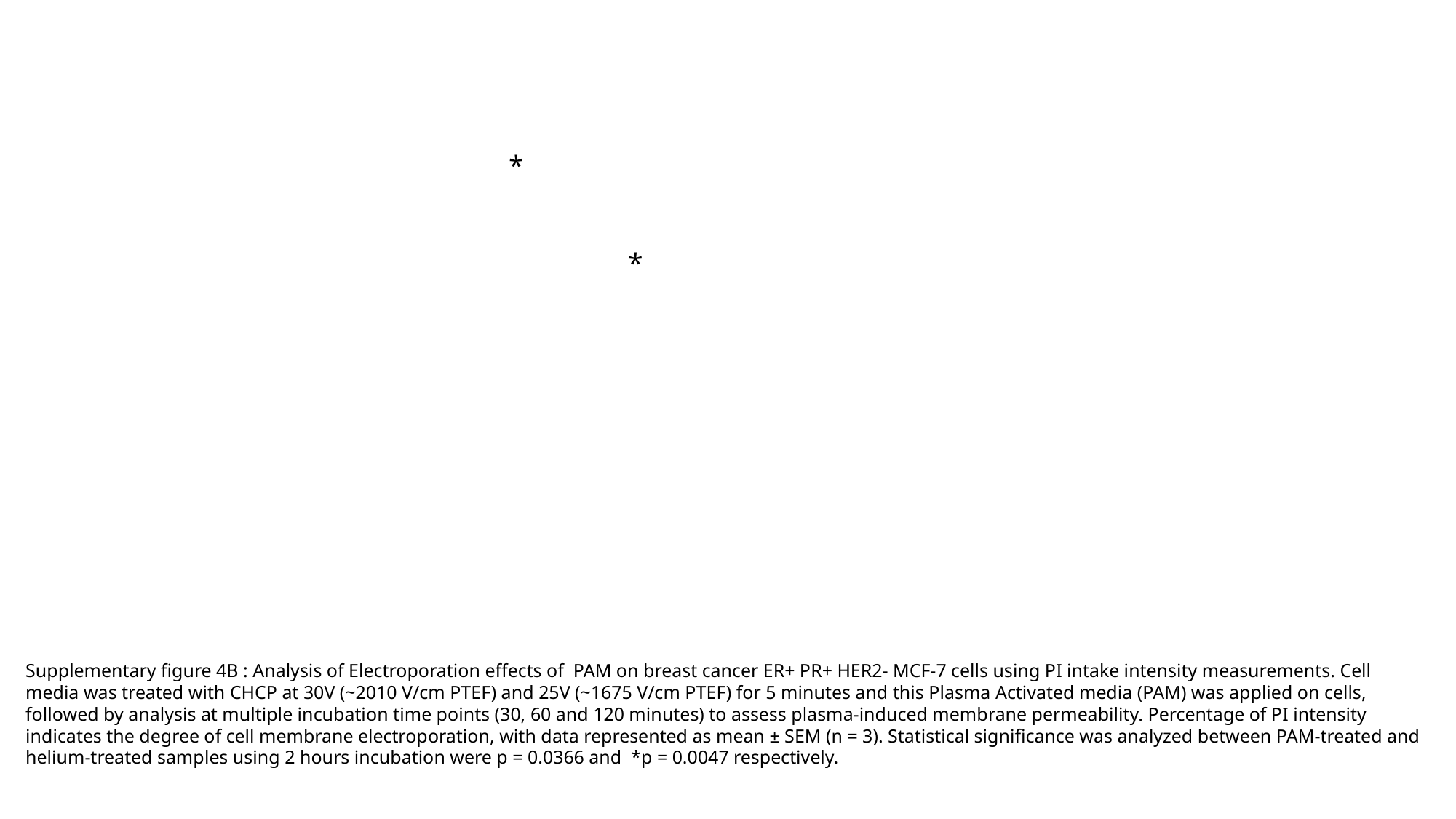

*
*
Supplementary figure 4B : Analysis of Electroporation effects of PAM on breast cancer ER+ PR+ HER2- MCF-7 cells using PI intake intensity measurements. Cell media was treated with CHCP at 30V (~2010 V/cm PTEF) and 25V (~1675 V/cm PTEF) for 5 minutes and this Plasma Activated media (PAM) was applied on cells, followed by analysis at multiple incubation time points (30, 60 and 120 minutes) to assess plasma-induced membrane permeability. Percentage of PI intensity indicates the degree of cell membrane electroporation, with data represented as mean ± SEM (n = 3). Statistical significance was analyzed between PAM-treated and helium-treated samples using 2 hours incubation were p = 0.0366 and *p = 0.0047 respectively.

## Slide 3
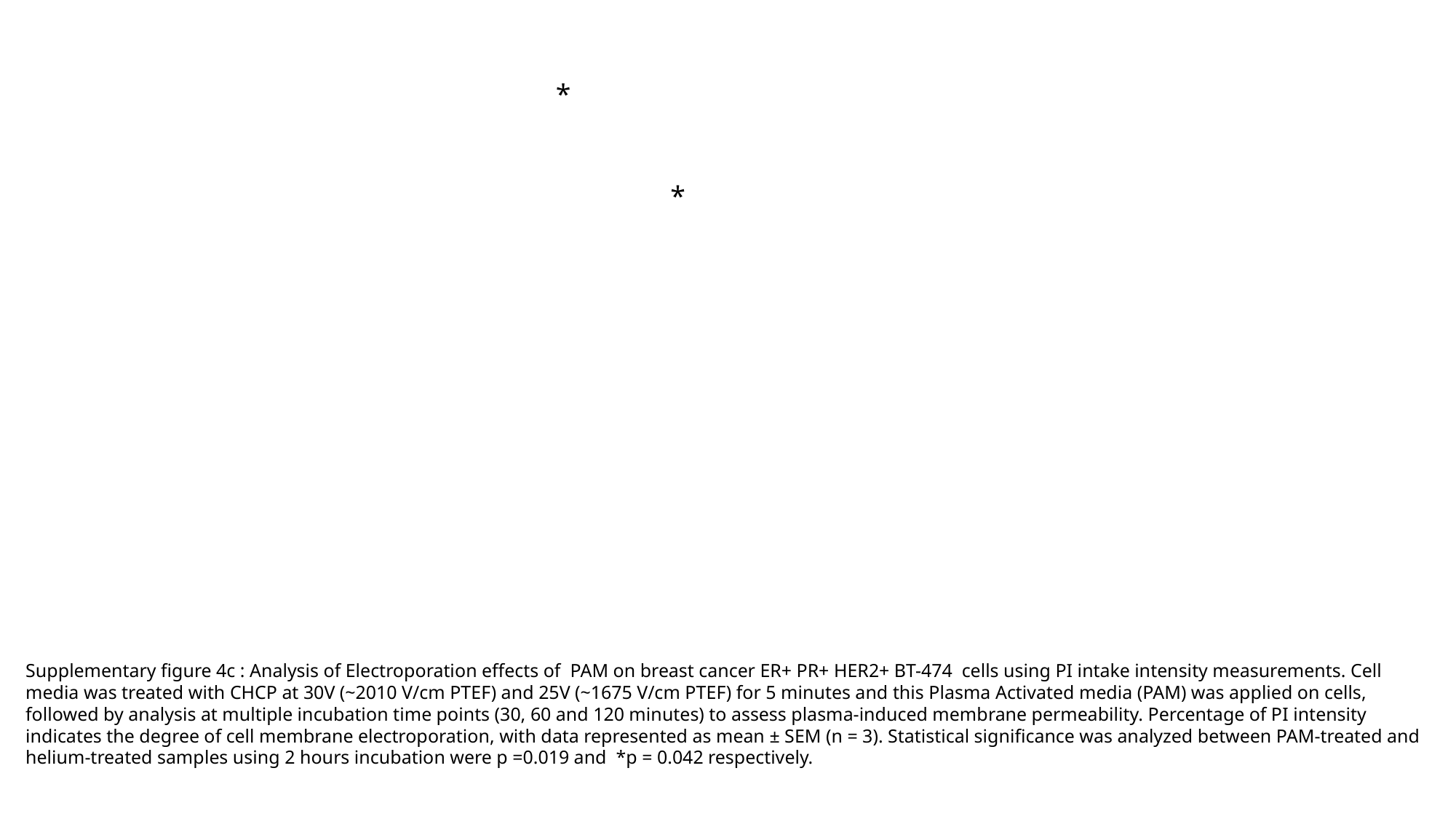

*
*
Supplementary figure 4c : Analysis of Electroporation effects of PAM on breast cancer ER+ PR+ HER2+ BT-474 cells using PI intake intensity measurements. Cell media was treated with CHCP at 30V (~2010 V/cm PTEF) and 25V (~1675 V/cm PTEF) for 5 minutes and this Plasma Activated media (PAM) was applied on cells, followed by analysis at multiple incubation time points (30, 60 and 120 minutes) to assess plasma-induced membrane permeability. Percentage of PI intensity indicates the degree of cell membrane electroporation, with data represented as mean ± SEM (n = 3). Statistical significance was analyzed between PAM-treated and helium-treated samples using 2 hours incubation were p =0.019 and *p = 0.042 respectively.
